# Supplementary material for: Community and single cell analyses reveal complex predatory interactions between bacteria in high diversity systems
Source: Nat Commun. 2021 Sep 16;12:5481. doi: 10.1038/s41467-021-25824-9 (PMC8446003; doi:10.1038/s41467-021-25824-9)
Supplement: Supplementary file 1 — Supplementary Information [file 41467_2021_25824_MOESM1_ESM.pdf]

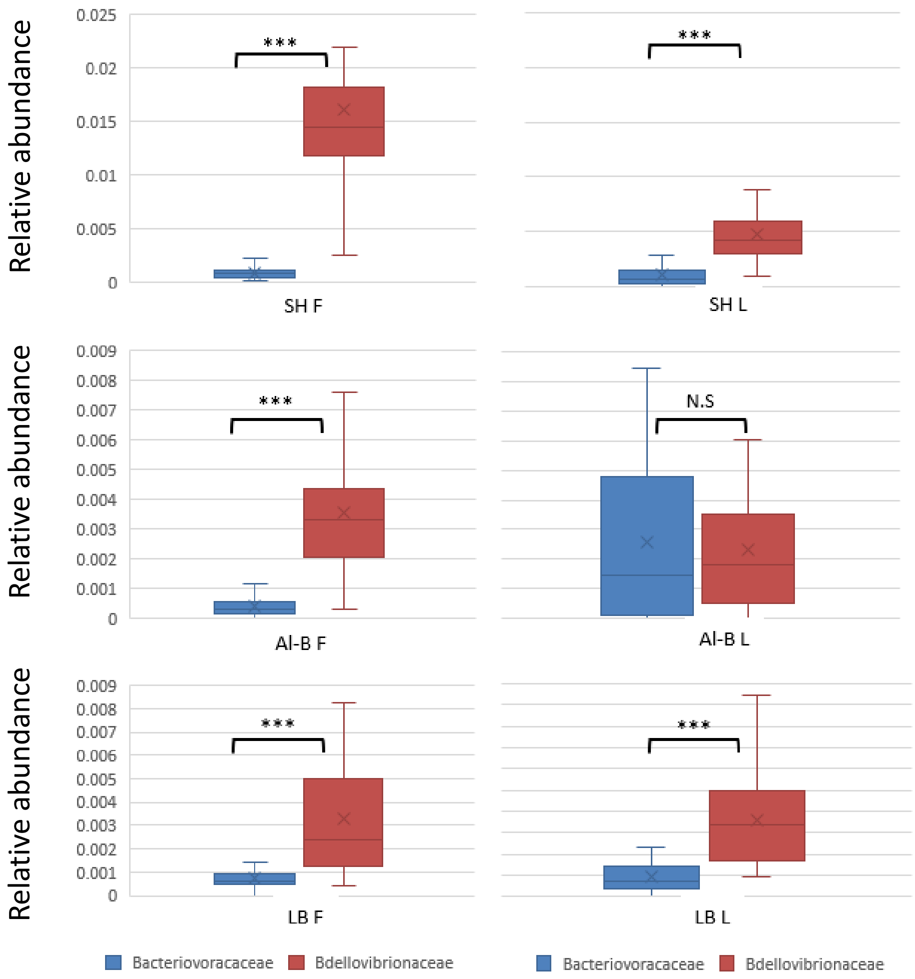

Figure S1. Relative abundance of BALOs. Box plot of the relative abundance in the total population of Bdellovibrionaceae (Bdellovibrionales) and Bacteriovoracaceae (Bacteriovoracales) 16S rRNA gene sequences from the floc (F) and liquor (L) fractions at the Shafdan (SH) and Al-Bireh (Al) and Langenreichenbach (LB) WWTPs, in a year long time series including 18 sampling times and a total of 198 samples. The analysis was based on a general bacterial 16S rRNA gene sequencing data of , and was performed at the family level. Box limits indicate the 25th and 75th percentiles. Whiskers show the standard deviation of the abundance distribution. Two-tailed t-test statistics on abundance distributions in Bdellovibrionaceae and Bacteriovoracaceae *** - p-value$<10^{-6}$, no adjustment for multiple comparisons.

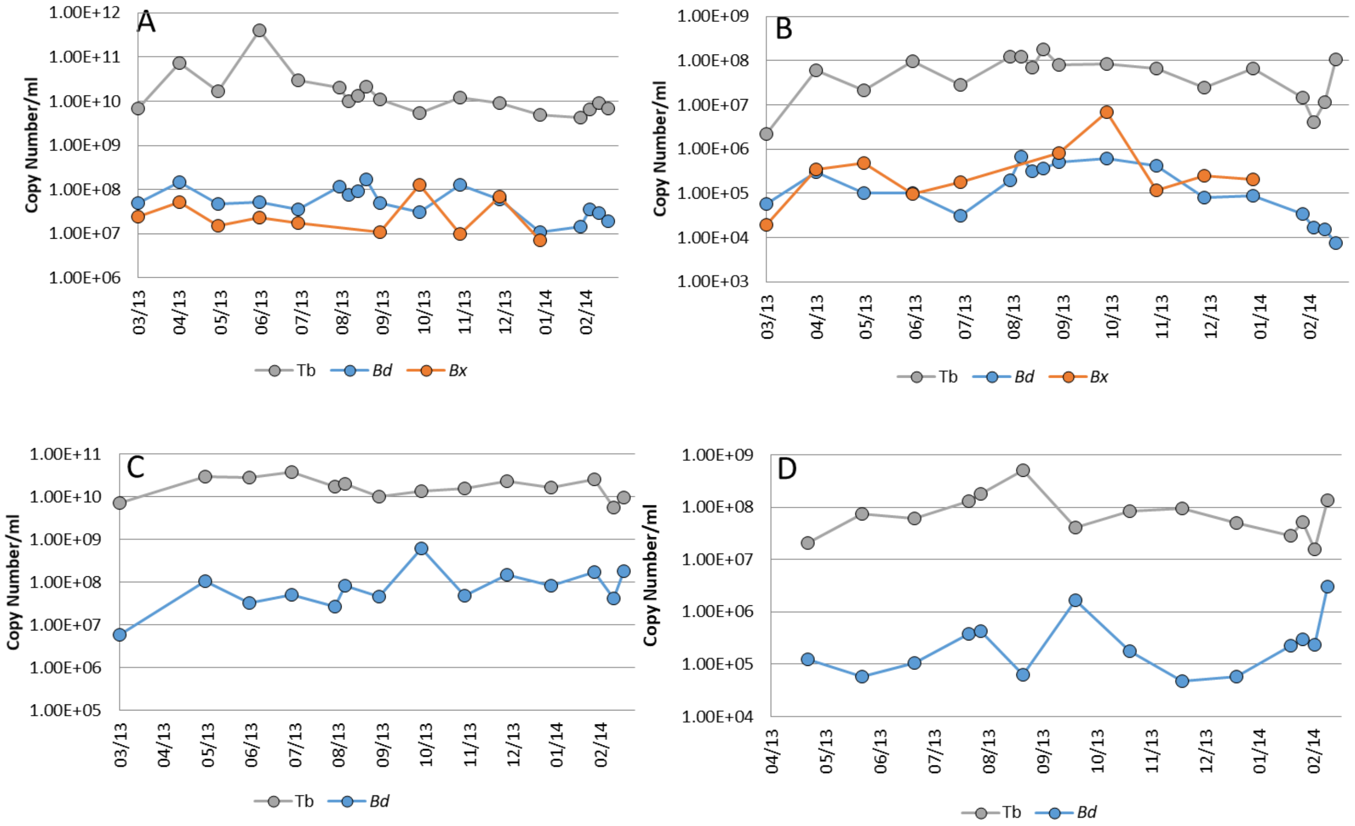

Figure S2. Quantitative PCR (QPCR) of BALO and general bacterial populations. QPCR-based analysis of the 16S rRNA gene dynamics of total bacteria (Tb, grey), Bdellovibrionales (Bd, blue) and Bacteriovoracales (Bx, ocher), in floc (A, C) and liquor (B, D) samples from the Shafdan (A, B), and Al-Bireh (C, D) WWTPs. Bd and Bx were measured at the Shafdan; only Bd was measured at Al-Bireh.

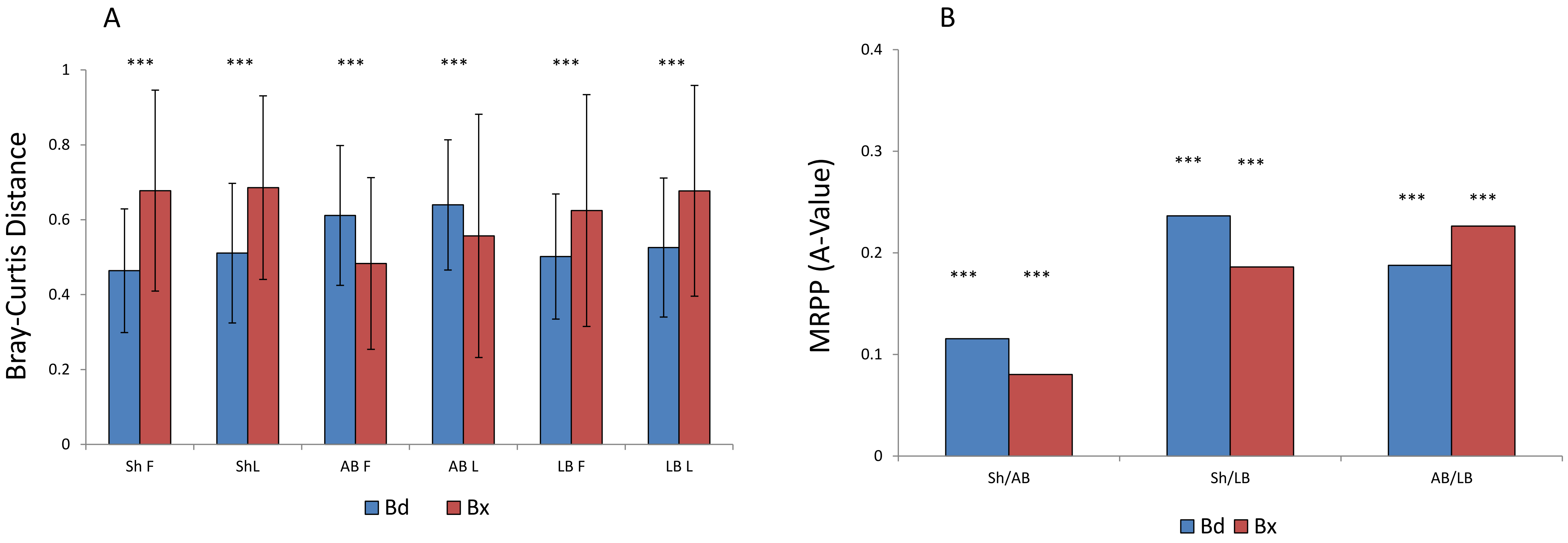

Figure S3. Distance-based analyses of BALO communities. A. Bray-Curtis distances of Bdellovibrionales (Bd) and Bacteriovoracales (Bx) communities within the WWTPs floc and liquor fractions. B. Dissimilarity values between the WWTPs for each community based on MRPP's A-value. Shafdan (SH), Al-Bireh (AB) and Langenreichenbach (LB). *** p-Value < 0.001. n=198 biological samples. P-values are based on two tailed Student's t-test, *** p-Value < 0.001 . Error bars show standard deviation, no adjustment for multiple comparisons.

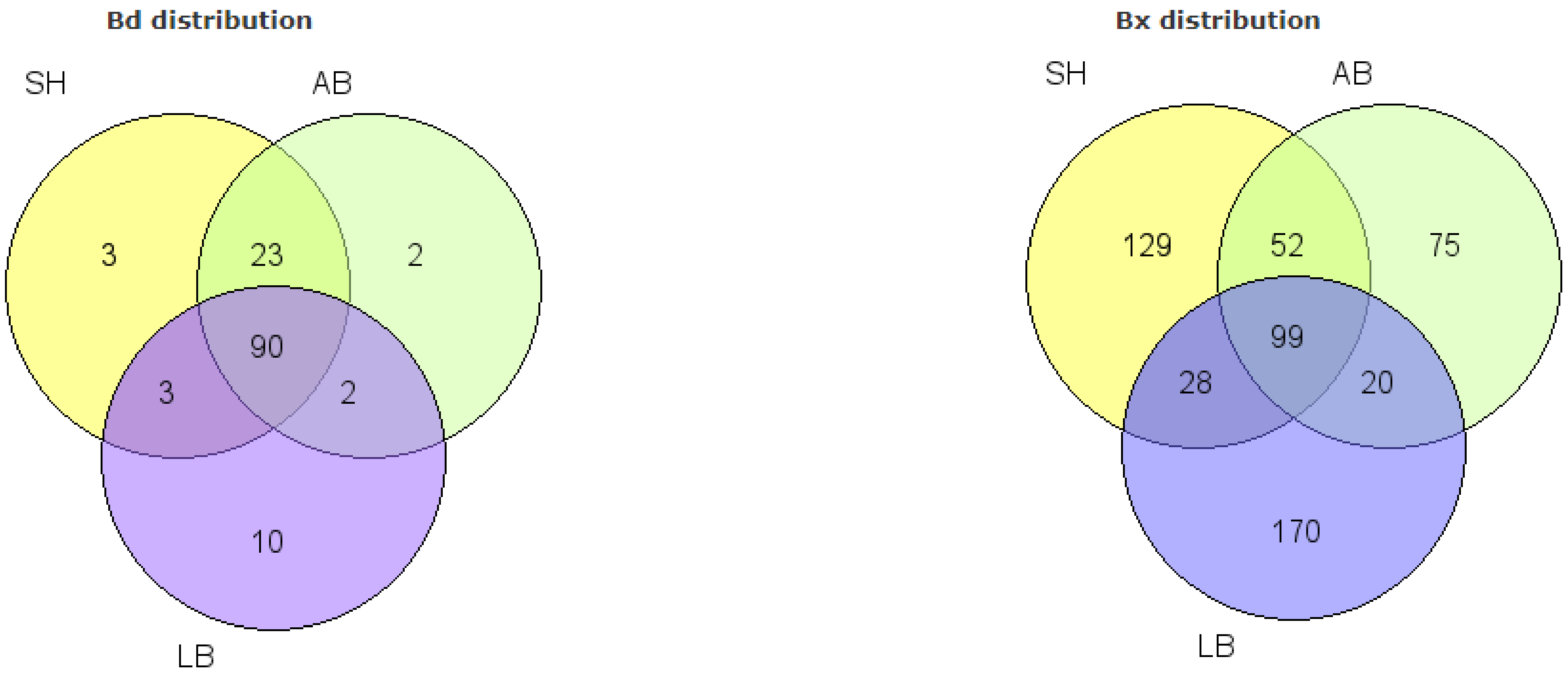

Figure S4. BALO distributions between the WWTPs. Common and shared Bdellovibrionales (Bd) and Bacteriovoracales (Bx) OTUs between the Shafdan (SH), Al-Bireh (AB) and Langenreichenbach (LB) WWTPs.

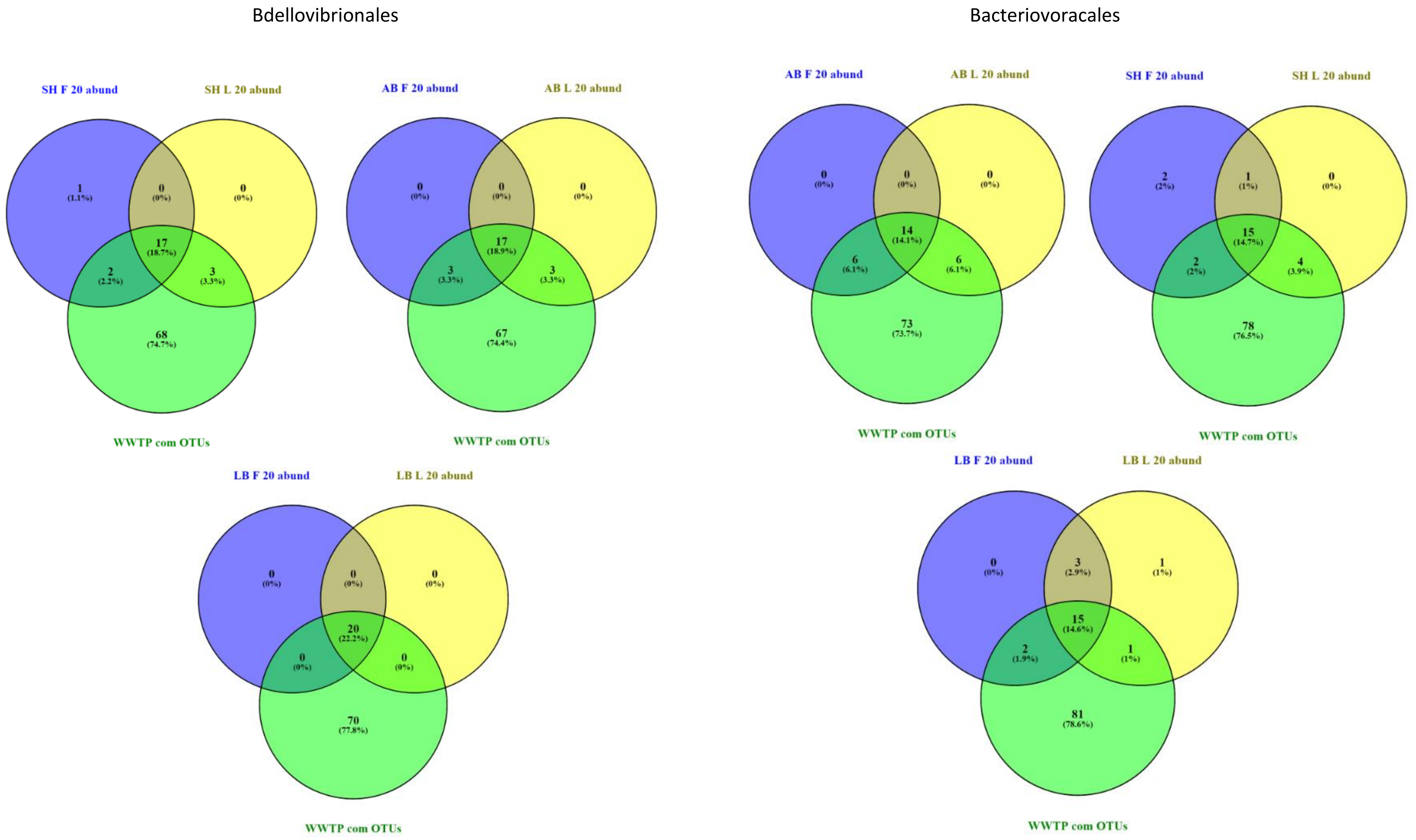

Figure S5. Distribution of most abundant BALOs. Overlap between the 20 most abundant Bdellovibrionales and Bacteriovoracales floc and liquor OTUs at the Shafdan (SH), Al-Bireh (AB) and Langenreichenbach (LB) WWTPs, and the OTUs common to all three WWTPs, in each clade.

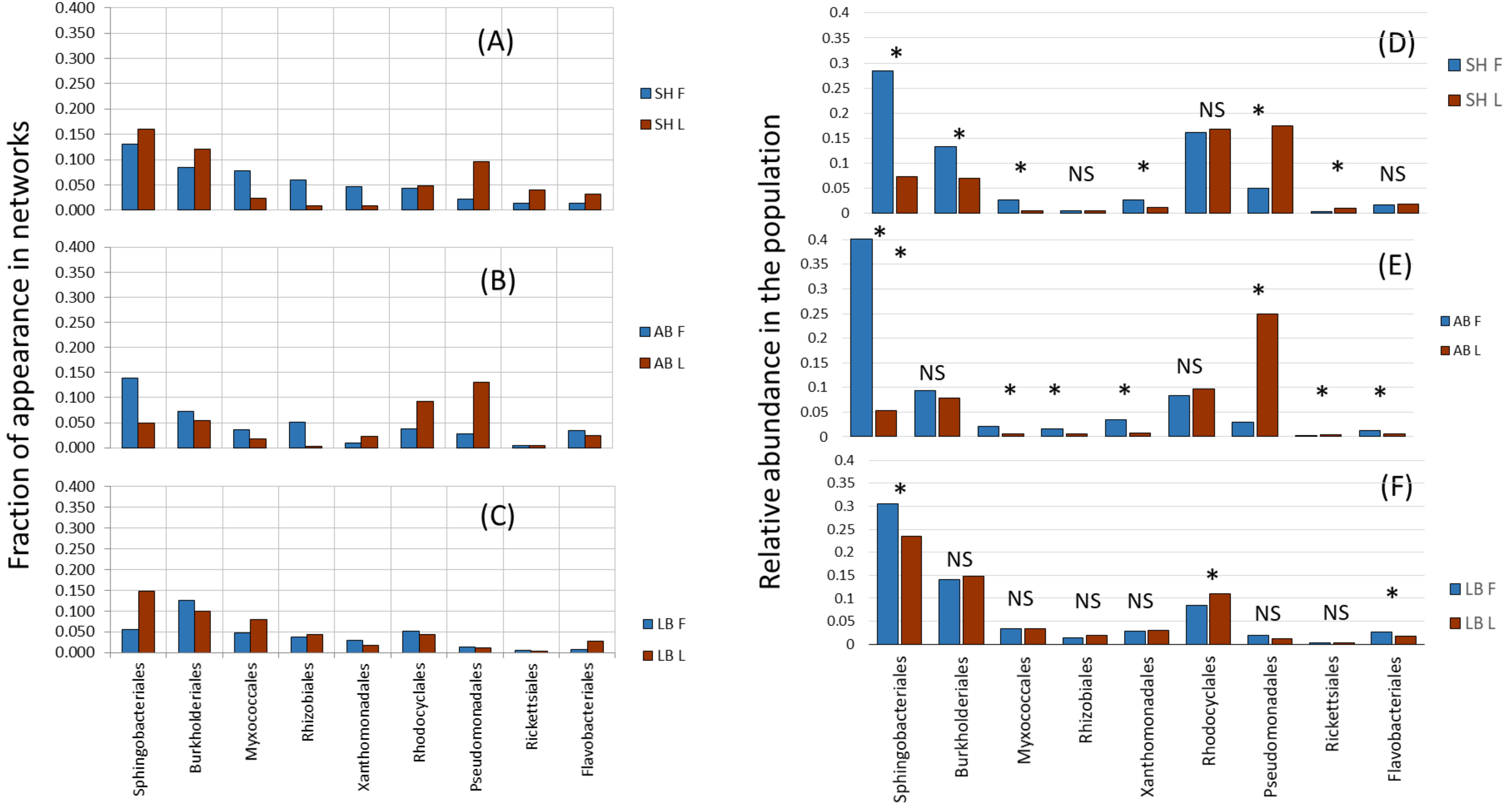

Figure S6. Most abundant prey OTUs. Fraction of the most abundant prey OTUs appearing in networks in the different WWTPs (A, B, C) and their relative abundance in the bacterial community (D, E, F), at each site and in each fraction. Shafdan (SH), Al-Bireh (AB), Langenreichenbach (LB); Flocs (F), liquor (L). Taxa are at the order level. t-test statistics on abundance distributions between flocs and liquor, $p<0.05$, NS –not significant, no adjustment for multiple comparisons).

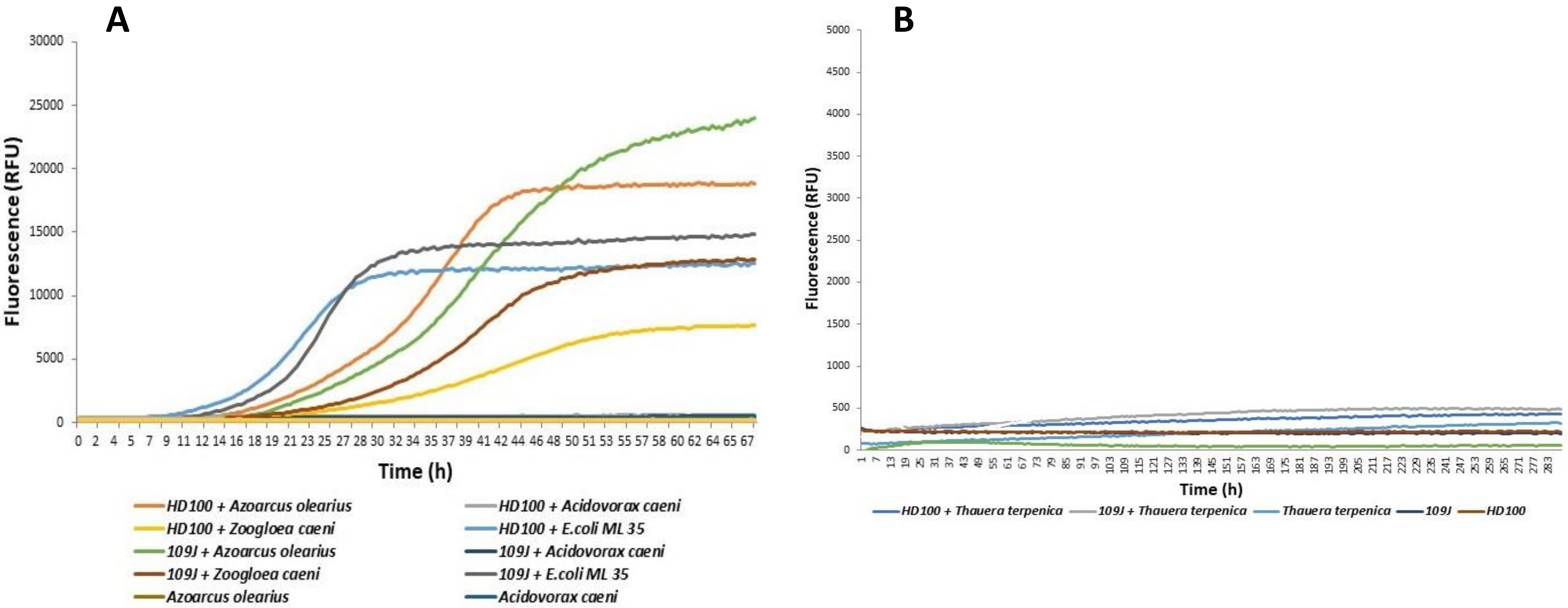

Figure S7. **BALO predation upon WWTP isolates**. Fluorescence-based tracking of the growth of Td-tomato protein-expressing *B. bacteriovorus* strains HD100 and 109J populations in the presence of prey strains (A) and non-prey strains (B), originating from WWTPs. *E. coli* ML35 is a reference prey control. Prey initial population density was circa 10^8 colony forming unit. ml^{-1} and predator initial population density circa 10^6 plaque forming units.m^{l-1}.

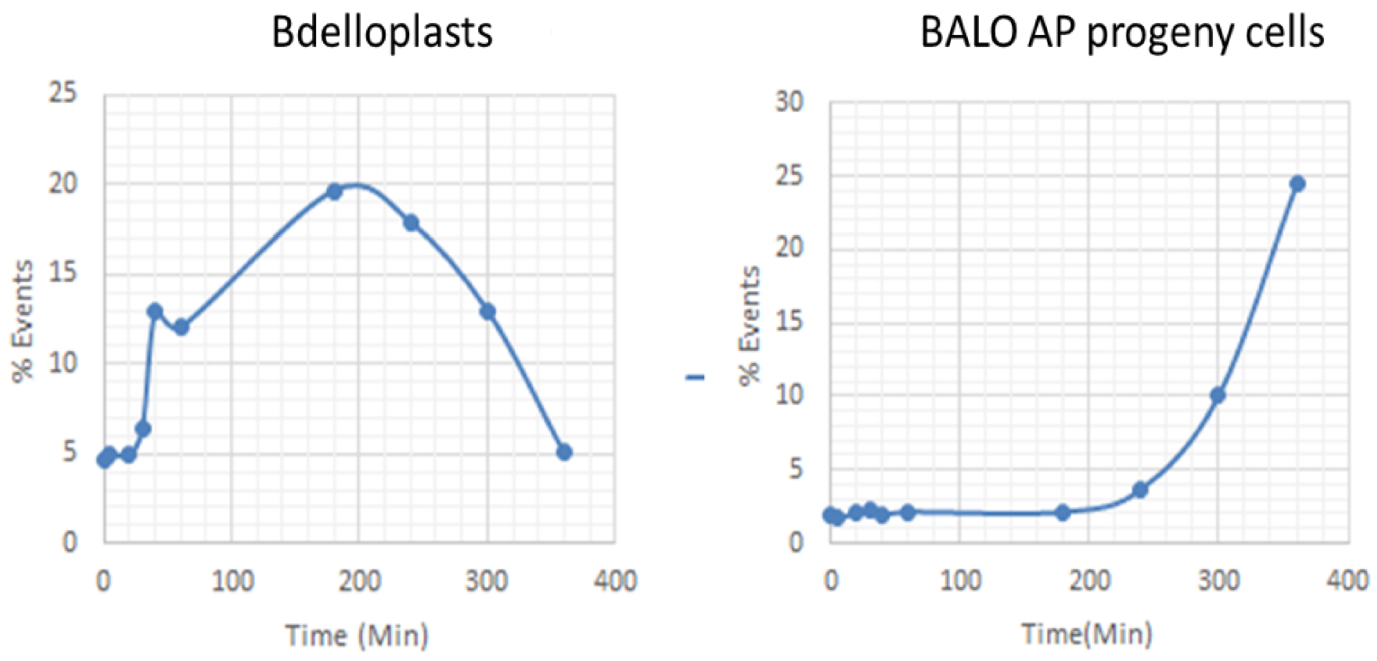

Figure S8. Flow cytometry analysis of a synchronous culture of *B. bacteriovorus* HD100 preying on *E. coli*. A. Dynamics of bdelloplast formation. B. Dynamics of progeny attack phase release.

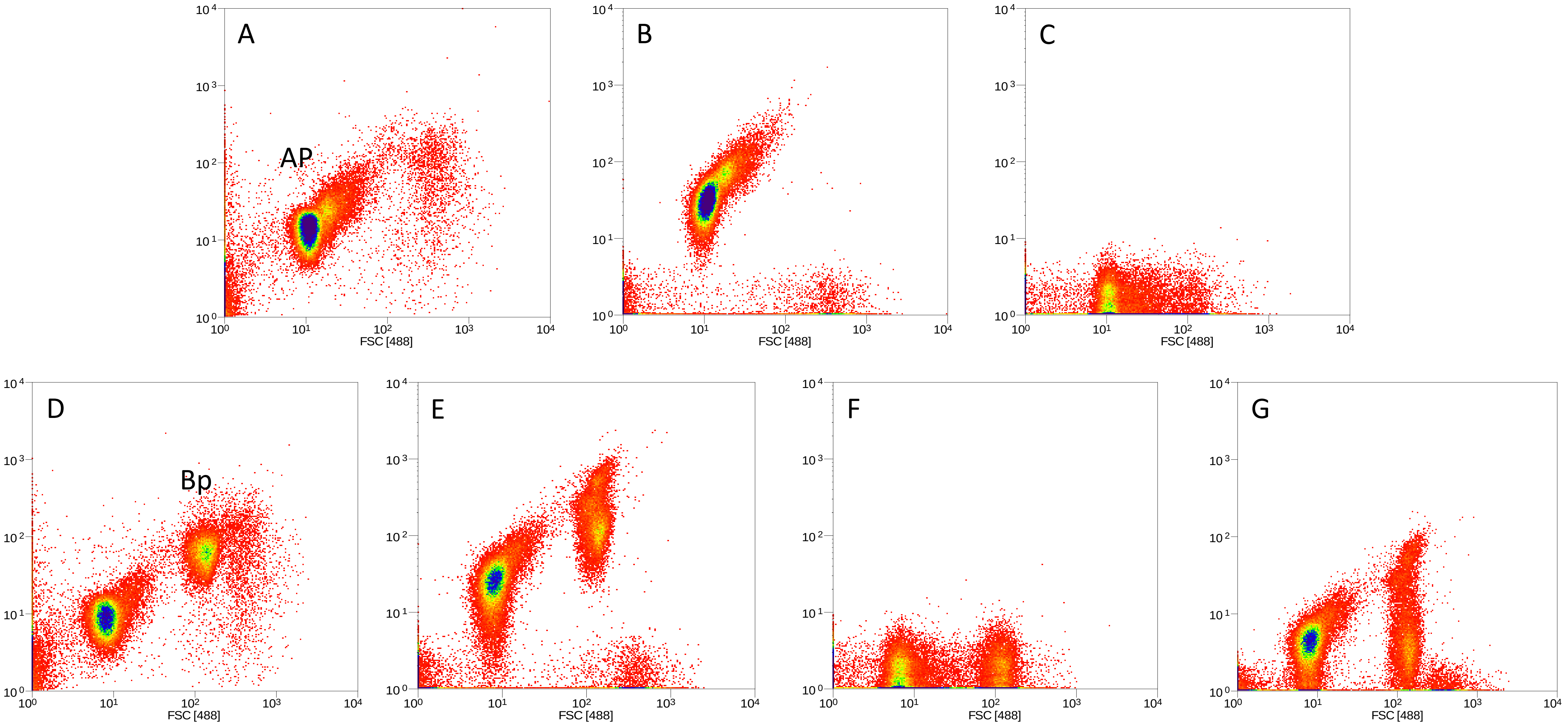

Figure S9. **Predator and prey flow cytometry (FC).** FC analysis of *B. bacteriovorus* HD100 attack phase (AP), and growth phase cells in bdelloplasts (Bp, *E. coli* prey). A. AP cells in the cell size vs. internal cell density (forward scatter (FSC)/side scatter (SSC)) detection mode; B. AP cells labelled with a BDE525-Cy5’ probe in the cell size vs. Cy5 fluorescence (FSC/fluorescence (670/30[640]) detection mode; C. AP cells labelled with a NonBDE525-Cy5’ probe in the cell size vs. Cy5 fluorescence (FSC/fluorescence (670/30[640]) detection mode; D. AP cells and Bp in the FSC/SSC mode; E. AP cells and Bp labelled with a BDE525-Cy5’ probe in the cell size vs. Cy5 fluorescence (FSC/fluorescence (670/30[640]) detection mode; F. AP cells and Bp labelled with a NonBDE-Cy5 in the cell size vs. Cy5 fluorescence (FSC/fluorescence (670/30[640]) detection mode and; G. AP cells and Bp labelled with a general bacterial EUB338-FITC probe in the cell size vs. FITC fluorescence (FSC/fluorescence (670/30[640]) detection mode.

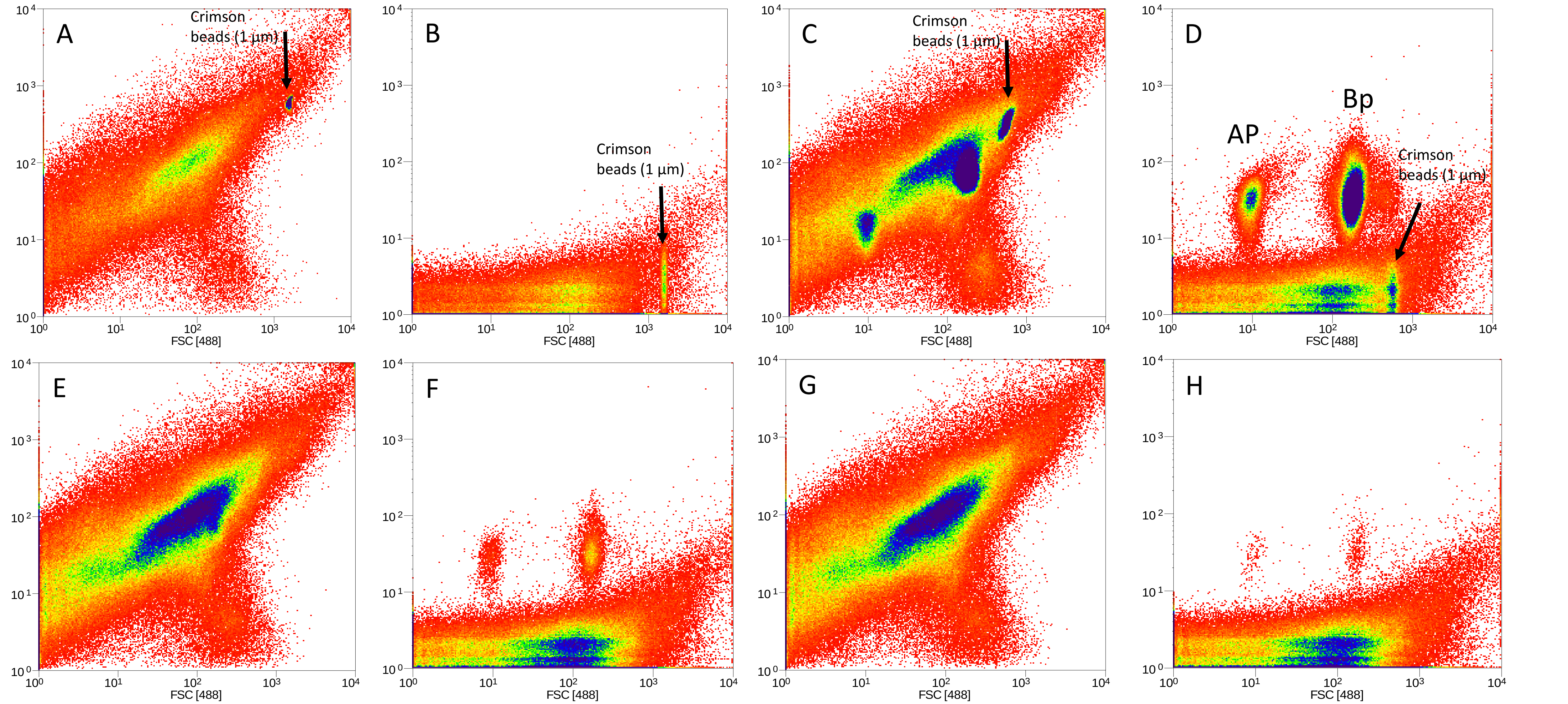

Figure S10. Flow cytometry (FC) of activated sludge (AS) sample spiked with BALOs. Attack (AP) cells and bdelloplasts (Bp) labelled with a BDE525-Cy5' probe, and control measurements were spiked in fixed AS samples A. Control AS sample in the forward scatter (FSC) /side scatter (SSC) detection mode; B. Control AS sample in the FSC/fluorescence (670/30[640]) (Fl) detection mode; AS sample spiked with (C) 10^8, (E) 10^7, and (G) 10^6 cells per ml (10, 1, 0.1% of total cell population), respectively of mixed AP cells and bdelloplasts labelled with BDE525-Cy5' in the FSC/SSC mode. AS sample spiked with (D) 10^8, (F) 10^7, and (H) 10^6 cells per ml (10, 1, 0.1% of total cell population), respectively of AP cells and bdelloplasts labelled with BDE525-Cy5' in the FSC/Fl detection mode. Crimson fluorescent beads (1 µm) were used for calibration (arrow)

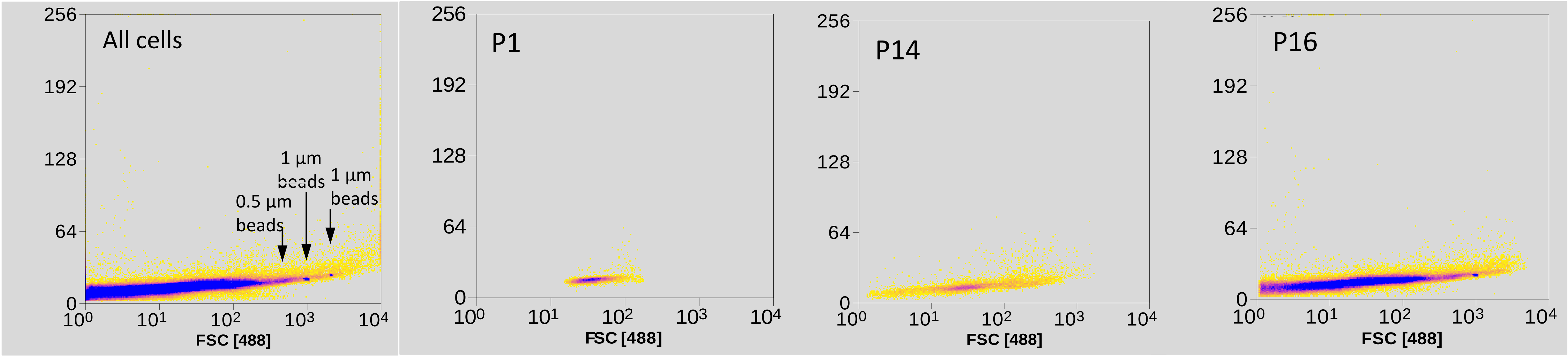

Figure S11. Flow cytometry (FC) quality control (QC). QC of the FC analysis by pulse width vs. forward scatter. All cells from wastewater sample, stained with DAPI and hybridized with a BDE525-Cy5' probe. P1: strongly fluorescent events (BDE525-Cy5' labelled cells); P14: weakly fluorescent events (BDE525-Cy5' labelled) cells; P16: non-fluorescent events (cells not labelled by the BDE525-Cy5' probe). The measurement validates single cell detection during the sorting process. More than 95% of the cells sorted from each gate were single cells.

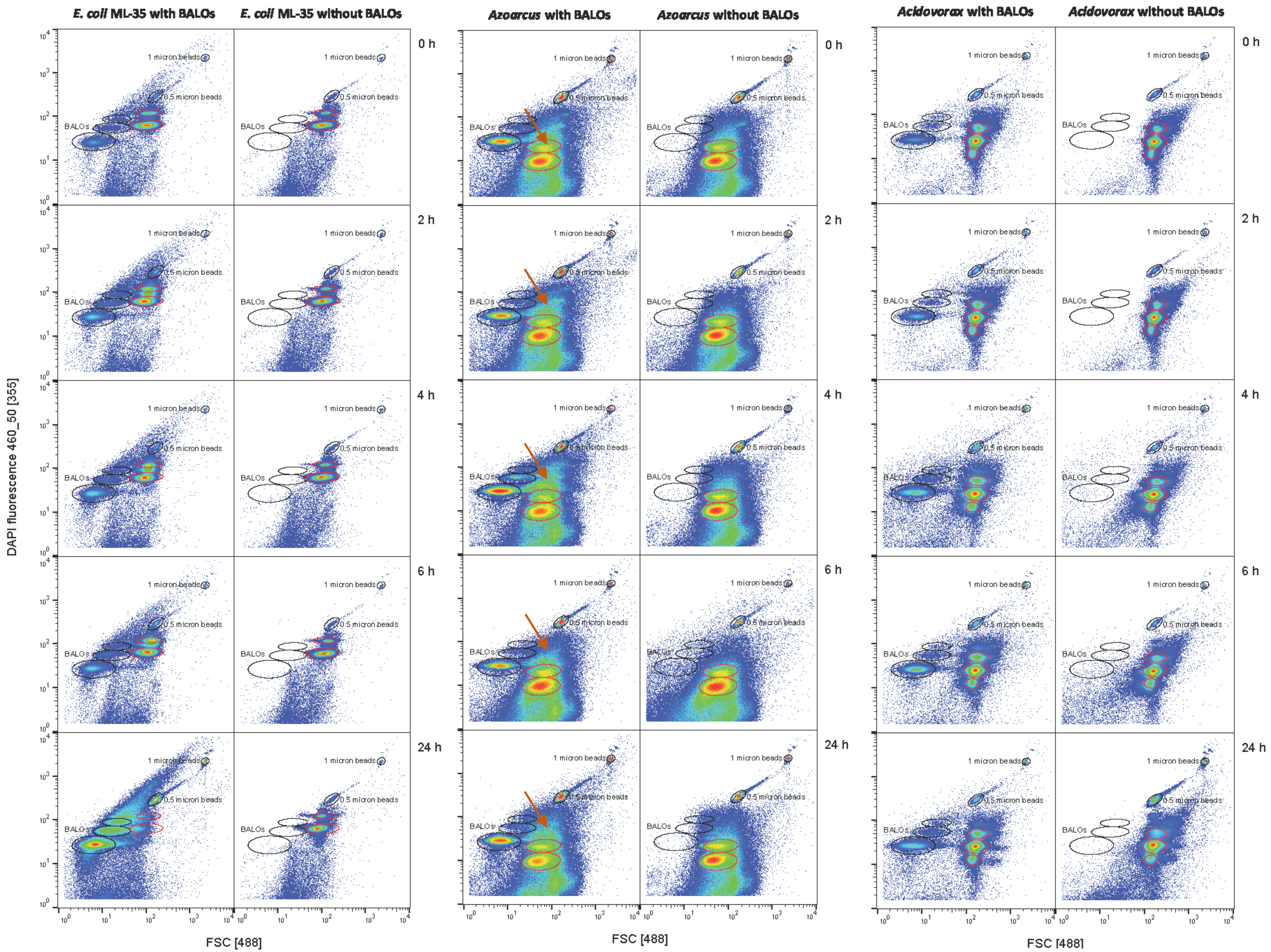

Figure S12. Flow cytometry analysis BALO-prey and BALO-non-prey interactions. Left: *Escherichia coli* ML-35 (prey) consumed by *B. bacteriovorus* HD100 within 24 h (empty red gates). Center: *Azoarcus* PA01 (prey) showed attachment of the predator (orange arrow), as compared to the *Azoarcus* PA01 alone. Right: *Acidovorax* M6 (non-prey) shows no variation in population patterns (unchanged red gates in the presence of the predator). Black and red gates indicate different subpopulations of the predator and of the tested strains, respectively. Upper right corner: 1 µm and 0.5 µm calibration beads.

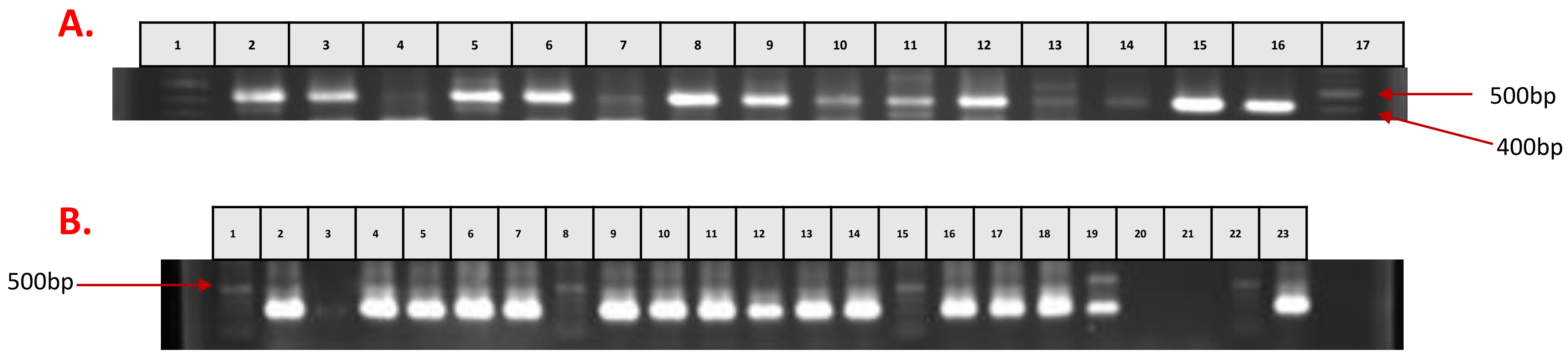

Figure S13. PCR validations of the sorted populations. A. PCR validation of the presence of *Bdellovibrio* spp. in gate P1 and gate P14 samples and of their absence from gate P16 samples, using *Bdellovibrio*-targeted 16S rRNA gene primers. Lanes: 1-Molecular weight marker, 2-P1, 3-P14, 4-P16, 5-P1, 6-P14, 7-P16, 8-P1, 9-P14, 10-P16, 11-P1, 12-P14, 13-P16, 14-Negative control, 15-Positive control (10^6 *B. bacteriovorus* cells), 16-Positive control (10^5 *B. bacteriovorus* cells), 17-Molecular weight marker; B. PCR amplification of DNA from gates P1, P14 and P16 samples using general bacteria-targeted 16S rRNA gene primers. Lanes: 1-Molecular weight marker, 2-P1, 3-P14 (failed reaction), 4-P16, 5-P1, 6-P14, 7-P16, 8-Molecular weight marker, 9-P1, 10-P14, 11-P16, 12-P1, 13-P14, 14-P16, 15-Molecular weight marker, 16-P1, 17-P14, 18-P16, 19-P14, 20-Negative control, 21-Negative control, 22-Molecular weight marker, 23-Positive control (*B. bacteriovorus* HD100). Each lane represents one sample.

Table S1. α-diversity estimates (S=Richness, E=Evenness, H=Shannon index, D'= Simpson index) of Bdellovibrionales and Bacteriovoracales communities calculated based on the grouping of 16S rDNA gene sequences as OTUs, obtained from 18 samples along a year, at the Shafdan (SH), Al-Bireh (AB), and Langenreichenbach (LB) WWTPs. F, floc fraction; L, liquor fraction. The S, E, H, and R values of Bdellovibrionales and Bacteriovoracales in each fraction at each WWTP are significantly different (t-test, p <0.01, two tailed). No differences were observed between fractions at each WWTP for the same family of predators, except between AB-F and AB-L.

		S	E	H	D`
Bdellovibrionaceae	**SH F**	77.4±7.6	0.57±0.1	2.48±0.5	0.82±0.1
	**SH L**	72.8±12.4	0.59±0.08	2.50±0.4	0.84±0.09
	**AB F**	69.7±6.9	0.57±0.09	2.42±0.4	0.83±0.1
	**AB L**	64.4±10.3	0.59±0.06	2.45±0.3	0.85±0.06
	**LB L**	56.4±9.0	0.63±0.06	2.53±0.2	0.86±0.06
	**LB F**	56.9±7.9	0.62±0.06	2.49±0.2	0.86±0.06
Bacteriovoracaceae	**SH F**	47±9.1	0.35±0.1	1.35±0.3	0.58±0.1
	**SH L**	51.6±11.7	0.38±0.1	1.51±0.4	0.59±0.2
	**AB F**	49.1±9.7	0.31±0.07	1.21±0.3	0.51±0.1
	**AB L**	40.4±6.3	0.30±0.1	1.09±0.4	0.45±0.2
	**LB L**	49.2±16.1	0.35±0.1	1.38±0.7	0.55±0.1
	**LB F**	47.8±15.1	0.40±0.2	1.52±0.6	0.59±0.3

Table S2. Pearson correlations (|r|) between the Bdellovibrionales and Bacteriovoracales predatory communities in the floc (F) and liquor (L) fractions at Shafdan (SH), Al-Bireh (AB) and Langenreichenbach (LB) WWTPs, and measured environmental parameters. Temperature (bold) (^{o}C); Salinity (ppm); conductivity (μS.m^{-1}); Total suspended solids (TSS) (mg.l^{-1}) Volatile suspended solids (VSS) (mg*l^{-1}); Biological oxygen demand (BOD, (mg.l^{-1}); Chemical oxygen demand (COD, (mg.l^{-1}); Nitrate (NO_2,ppm); Nitrate (NO_3,ppm); Phosphorus (PO_4,ppm); Total Kjehldahl nitrogen (TKN, mg.l^{-1}); Total organic carbon (TOC, mg.l^{-1}); Total dissolved solids (TDS, mg.l^{-1}). In bold – Pearson >0.5.

		SH F	SH L	AB F	AB L	LB F	LB L
Bdellovibrionaceae	Temp[°C]	**0.789**	**0.705**	**0.46**	**0.626**	**0.566**	**0.507**
	Salinity	0.175	0.34	0.378	0.132	0.226	0.569
	pH	0.384	0.174	0.191	0.03	0.158	0.426
	Conductivity	0.261	0.212	0.298	0.164	0.055	0.181
	TSS(mg/l	0.055	0.267	0.134	0.565	0.622	0.099
	VSS(mg/l)	0.048	0.26	0.147	0.412	0.139	0.129
	BOD(mg/l	0.02	0.268	0.392	0.197	0.403 (CBOD5Re)	0.049 (CBOD5Re)
	COD(mg/l	0.006	0.039	0.229	0.272		
	NO2	0.249	0.157	0.262	0.586	0.297	0.538
	NO3(ppm)	0.28	0.353	0.377	0.386	0.281	0.295
	NH4(ppm)	0.248	0.345	0.068	0.24	0.146	0.273
	PO4(ppm)	0.037	0.378	0.482	0.065	0.098	0.473
	TKN	0.456	0.647	0.08	0.151	0.188	0.608
	TOC	0.347	0.339	0.135	0.276	0.091	0.085
	TDS(mg/l	0.372	0.171	0.216	0.404	N/A	N/A
Bacteriovoracaceae	Temp[°C]	**0.95**	**0.919**	**0.221**	**0.536**	**0.785**	**0.804**
	Salinity	0.119	0.065	0.376	0.021	0.32	0.61
	pH	0.411	0.403	0.003	0.362	0.102	0.756
	Conductivity	0.604	0.694	0.368	0.016	0.239	0.055
	TSS(g/l)	0.208	0.179	0.011	0.567	0.605	0.006
	VSS(g/l)	0.214	0.284	0.46	0.236	0.012	0.249
	BOD(mg/l	0.287	0.634	0.029	0.157	0.392 (CBOD5)	0.069 (CBOD5)
	COD(mg/l	0.314	0.403	0.266	0.019		
	NO2	0.036	0.25	0.567	0.127	0.055	0.468
	NO3(ppm)	0.005	0.519	0.599	0.881	0.504	0.429
	NH4(ppm)	0.015	0.009	0.279	0.541	0.205	0.461
	PO4(ppm)	0.335	0.438	0.653	0.052	0.046	0.092
	TKN	0.228	0.67	0.429	0.305	0.327	0.652
	TOC	0.096	0.29	0.638	0.759	0.122	0.216
	TDS(mg/l	0.009	0.051	0.164	0.299	N/A	N/A

Table S3. Parameters describing the bacterial predator-prey networks derived from negative (-0.7>) and significant ($p<0.05$) Kendall correlations. Edges are the number of predator-prey interactions in each sludge fraction (floc/liquor). Edges/Connections (%) indicates the percentage of positive edges out of the total possible number of connections. Nodes are the number of predators and prey found to have positive associations according to the criteria above. Average degree/ node connectivity is the average number of edges that are incident on a node. Modularity Index values > 0.4 suggest the network has a modular structure (hence more robust). Averaged edges of Bdellovibrionales (Bd), and of Bacteriovoracales (Bx) are the average number of connections with prey for each of the two families. Shared predators/prey indicate the ratio (in %) of OTUs which appear both in the floc and in the liquor at each WWTP.

	**SH F**	**SH L**	**AB F**	**AB L**	**LB F**	**LB L**
**Bacterial OTUs**	3374	3468	3528	3601	3778	3833
**Bd OTUs**	111	114	112	110	102	99
**Bx OTUs**	219	240	205	181	235	250
**Total no. of connections**	1106672	1133650	1112636	1042944	1257648	1228539
**Edges (<=-0.7)**	292	113	516	830	399	537
**Edges/ Connections (%)**	0.026	0.010	0.046	0.080	0.032	0.044
**Nodes (Predator) (Bd/Bx)**	70 (44/26)	49 (32/17)	67 (41/26)	77 (51/26)	65 (36/29)	83 (41/42)
**Nodes (Prey)**	152	70	243	336	232	299
**Average degree/ node connectivity**	2.69	1.65	3.03	3.82	2.61	2.71
**Network Diameter**	13	12	10	10	12	14
**Averaged Path Length**	4.28	4.49	3.93	3.83	4.62	4.50
**Modularity Index**	0.73	0.86	0.67	0.54	0.77	0.74
**Avg. Edges for Bd (±SE)**	4.52 ±0.76	2.41 ±0.32	9.24 ±1.80	8.61 ±2.07	6.67 ±0.89	6.71 ±1.52
**Avg. Edges for Bx (±SE)**	3.57 ±0.61	2.18 ±0.41	5.26 ±1.21	15.04 ±3.05	5.48 ±1.02	6.24 ±1.08
**Shared Predators (%)**	48.8		41.2		43.7	
**Shared Prey (%)**	13.5		12.1		14.7	

Table S4. Characteristics of the wastewater treatment plants (WWTP) sampled in this study. A. Location and size of the plants. B. Average values (± SD) of chemical parameters measured in the floc (F) and liquor (L) fractions at Shafdan (SH), Al-Bireh (AB) and Langenreichenbach (LB). Temperature (°C); Salinity (ppm); conductivity (µS/m); Total suspended solids(TSS)(mg.l^{-1}) Volatile suspended solids (VSS) (mg.l^{-1}); Biological oxygen demand (BOD, (mg.l^{-1}); Chemical oxygen demand (COD, (mg.l^{-1}); Nitrate (NO_2,ppm); Nitrate (NO_3,ppm); Phosphorus (PO_4,ppm); Total Kjehldahl nitrogen (TKN, mg.l^{-1}); Total organic carbon (TOC, mg.l^{-1}); Total dissolved solids (TDS, mg.l^{-1}).

A.

WWTP	Details
Shafdan, Israel	A central WWTP serving a population of two millions in the Dan region. Treats 130 million cubic meters of wastewater annually.
Al-Bireh, Palestine	A communal WWTP. Treats wastewater for a population of 45000-50000, including industrial wastewater.
Langenreichenbach, Germany	A communal WWTP (no industrial wastewater). Located 50km northeast to Leipzig, Treats wastewater from 4000 homes.

B.

	Temp [°C]	Salinity	pH	Conductivity	TSS	VSS	COD	BOD	NO_2	NO_3	NH_4	PO_4	TKN	TOC	TDS
Sh F	25.3 ±3.9	0.63 ±0.1	7.6 ±0.4	1281.3 ±185.5	2459.9 ±1005.4	2223.6 ±937.9	4099.2 ±15.1	2365.7 ±15.4	2.63 ±2.4	5.7 ±3.3	4.9 ±2.6	26.7 ±16.1	10.1 ±4.1	19.8 ±15.7	786.2 ±104.1
Sh L	25.4 ±3.9	0.64 ±0.06	7.6 ±2.9	1303.8 ±141.6	11.7 ±15.3	8.1 ±1.2	30.2 ±9.2	12.5 ±3.9	3.3 ±2.3	8.6 ±3.8	3.6 ±2.4	11.3 ±6.2	5.4 ±4.3	19.3 ±16.8	816.5 ±110.9
AB F	21.2 ±7.4	0.56 ±0.1	7.0 ±0.2	1455.1 ±213.6	3285.33 ±1197.9	4362.9 ±1971.5	4774.2 ±1575.2	1110.7 ±436.6	0.26 ±0.2	44.9 ±37.9	6.6 ±7.0	18.0 ±20.9	N/A	14.2 ±10.0	895.1 ±145.4
AB L	21.9 ±7.1	0.57 ±0.2	7.4 ±0.3	1413.3 ±191.7	25.7 ±34.057	3984.2 ±1601.8	119.9±1 54.1	43.52 ±24.3	0.19 ±0.1	59.3 ±53.6	4.1 ±4.6	2.8 ±2.8	N/A	12.5 ±7.6	855.2 ±132.4
LB F	14.8 ±5.0	0.66 ±0.1	6.8 ±0.0	984.7 ±282.6	4338.9 ±981.6	1353.7 ±250.9	2210.8±847.3 (CBOD5 Re)		0.16 ±0.1	16.4 ±7.7	3.3 ±10.1	1.0 ±0.2	9.4 ±1.2	3752.0 ±796.1	N/A
LB L	14.3 ±5.6	0.71 ±0.2	6.9 ±1.6	1043.1 ±290.3	2.0 ±1.7	0.61 ±0.5	709.7±1097.1 (CBOD5 Re)		0.07 ±0.1	18.0 ±6.3	0.9 ±1.8	1.1 ±0.2	7.1 ±4.0	16.8 ±14.3	N/A

Table S5. Probes and primers used in this study.

Name	Sequence (5’-3’)	Position	%GC	Tm	Labels	Reference	
BbsF216	TTTCGCTCTAAGATGAGTCCGCGT	216-239	50	57.4		Essche et al. 2009	16S qPCR Bdellovibrionaceae primers
BbsR707	TTCGCCTCCGGTATTCCTGTTGAT	683-707	50	57.4		Essche et al. 2009	16S qPCR Bdellovibrionaceae primers
Bd347F	GGAGGCAGCAGTAGGGAATA	347–366	55	56.2		Essche et Al. 2009	16S qPCR Bdellovibrionaceae primers
Bd549R	GCTAGGATCCCTCGTCTTACC	529–549	57.1	56.4		Essche et al. 2009	16S qPCR Bdellovibrionaceae primers
BacF69	TGGCGCACGGGCTGAGTAAC	69-88	65	57.9		This study	16S qPCR Bacteriovoracaceae primers
BacR1049	AGCCATGCAGCGCCTGTCAC	1030-1049	65	57.9		This study	16S qPCR Bacteriovoracaceae primers
BacF519	CAGCAGCCGCGGTAATAC	519-536	61	57.47		Zheng et al. 2008	16S Bacteriovoracaceae qPCR primers
BacR677	CGGATTTTACCCCTACATGC	658-677	50	57.35		Zheng et. Al. 2008	16S Bacteriovoracaceae qPCR primers
BDE525*	GATCCCTCGTCTTACCGC	525-542	61	58.4	5' Cy3	Mahmoud et al. 2007	16S Bdellovibrionaceae FISH probe
Univ16S-1	GTGSTGCAYGGYTGTCGTCA	1048-1067	50	53.8-57.9		Nakatsuji et al. 2013	16S Bacteria Universal qPCR primers
Univ16S-1	ACGTCRTCCMCACCTTCCTC	1156-1175	55	53.8-57.9		Nakatsuji et al. 2013	16S Bacteria Universal qPCR primers
Bd824F	ACTTGTTGTTGGAGGTAT	824-841	39	43.5		Pasternak et al. 2013	16S rRNA gene primers of Bdellovibrionales
Bd1222R	TTGTAGCACGTGTGTAG	1206-1222	47	44.6		Pasternak et al. 2013	16S rRNA gene primers of Bdellovibrionales
Bx341F	CTACGGGAGGCAGCAG	341-355	69	51,1		Pasternak et al. 2013	16S rRNA gene primers of Bdellovibrionales
Bx672R	TACCCCTACATGCGAAATTCC	652-672	48	52.4		Pasternak et al. 2013	16S rRNA gene primers of Bdellovibrionales
515F	GTGCCAGCMGCCGCGGTAA	515-533	68	57.6-59.7		Walters et al. 2016	16S rRNA gene primers of Total Bacteria
926R	CCGYCAATTYMTTTRAGTTT	906-926	25	41.5-49.7		Walters et al. 2016	16S rRNA gene primers of Total Bacteria

Supplementary References

Mahmoud, K. K., McNeely, D., Elwood, C., & Koval, S.F. Design and performance of a 16S rRNA-targeted oligonucleotide probe for detection of members of the genus *Bdellovibrio* by fluorescence in situ hybridization. *Appl. Environ. Microbiol.* **73**, 7488-7493, doi:10.1128/aem.01112-07 (2007)

Nakatsuji, T. *et al.* The microbiome extends to subepidermal compartments of normal skin. *Nat. Commun.* **4**, 1431, doi:10.1038/ncomms2441 (2013)

Van Essche, M. et al. Development and performance of a quantitative PCR for the enumeration of Bdellovibrionaceae. Environ. Microbiol. Rep. 1, 228-233, doi:10.1111/j.1758-2229.2009.00034.x (2009).

Walters, W. et al. Improved bacterial 16S rRNA gene (V4 and V4-5) and fungal internal transcribed spacer marker gene primers for microbial community surveys. Msyst. 1;1(1):e00009-15 (2016)

Zheng, G., Wang, C, Williams, HN, & Pineiro, SA. Development and evaluation of a quantitative real-time PCR assay for the detection of saltwater *Bacteriovorax*. *Environ. Microbiol.* **10**, 2515-2526 (2008).
