# Supplementary material for: Community and single cell analyses reveal complex predatory interactions between bacteria in high diversity systems
Source: Nat Commun. 2021 Sep 16;12:5481. doi: 10.1038/s41467-021-25824-9 (PMC8446003; doi:10.1038/s41467-021-25824-9)
Supplement: Supplementary file 3 — Description of Additional Supplementary Files [file 41467_2021_25824_MOESM3_ESM.pdf]

Description of Additional Supplementary Files

File Name: Supplementary Data 1
Description: Taxonomic affiliations of predators and of their prey.

File Name: Supplementary Data 2
Description: Predators prey range in flocs and liquor.

File Name: Supplementary Data 3
Description: Prey OTUs detected both in the P1 and P 14 gates in the sorting experiment, and in the networks constructed with the Langenreichenbach WWTP dataset (Figure 5 E, F), using a sequence similarity threshold of 97% and an E value<1E-50.
