# Supplementary material for: Community and single cell analyses reveal complex predatory interactions between bacteria in high diversity systems
Source: Nat Commun. 2021 Sep 16;12:5481. doi: 10.1038/s41467-021-25824-9 (PMC8446003; doi:10.1038/s41467-021-25824-9)
Supplement: Supplementary file 7 — Reporting Summary [file 41467_2021_25824_MOESM7_ESM.pdf]

nature portfolio

Corresponding author(s): Edouard Jurkevitch

Last updated by author(s): 2021/08/25

Reporting Summary

Nature Portfolio wishes to improve the reproducibility of the work that we publish. This form provides structure for consistency and transparency in reporting. For further information on Nature Portfolio policies, see our Editorial Policies and the Editorial Policy Checklist.

Statistics

For all statistical analyses, confirm that the following items are present in the figure legend, table legend, main text, or Methods section.

n/a	Confirmed	
☐	☒	The exact sample size (n) for each experimental group/condition, given as a discrete number and unit of measurement
☐	☒	A statement on whether measurements were taken from distinct samples or whether the same sample was measured repeatedly
☐	☒	The statistical test(s) used AND whether they are one- or two-sided<br>*Only common tests should be described solely by name; describe more complex techniques in the Methods section.*
☐	☒	A description of all covariates tested
☒	☐	A description of any assumptions or corrections, such as tests of normality and adjustment for multiple comparisons
☐	☒	A full description of the statistical parameters including central tendency (e.g. means) or other basic estimates (e.g. regression coefficient) AND variation (e.g. standard deviation) or associated estimates of uncertainty (e.g. confidence intervals)
☐	☒	For null hypothesis testing, the test statistic (e.g. F, t, r) with confidence intervals, effect sizes, degrees of freedom and P value noted<br>*Give P values as exact values whenever suitable.*
☒	☐	For Bayesian analysis, information on the choice of priors and Markov chain Monte Carlo settings
☐	☒	For hierarchical and complex designs, identification of the appropriate level for tests and full reporting of outcomes
☐	☒	Estimates of effect sizes (e.g. Cohen's d, Pearson's r), indicating how they were calculated

Our web collection on statistics for biologists contains articles on many of the points above.

Software and code

Policy information about availability of computer code

Data collection	*Provide a description of all commercial, open source and custom code used to collect the data in this study, specifying the version used OR state that no software was used.*
Data analysis	Mother v,1.4; Silva v.132; PC-ORD v6.0; iTol V.4; genevenn has no updates; Gephi v.0.9.2i; FlowJo v10.0.8.r.1; R package flow Cybar v 3.13; R package flowCHIP v 3.13

For manuscripts utilizing custom algorithms or software that are central to the research but not yet described in published literature, software must be made available to editors and reviewers. We strongly encourage code deposition in a community repository (e.g. GitHub). See the Nature Portfolio guidelines for submitting code & software for further information.

Data

Policy information about availability of data

All manuscripts must include a data availability statement. This statement should provide the following information, where applicable:

- Accession codes, unique identifiers, or web links for publicly available datasets
- A description of any restrictions on data availability
- For clinical datasets or third party data, please ensure that the statement adheres to our policy

Source data are available in the supplementary material. All row flow cytometry data are deposited in the FlowRepository database (www. Flowrepository.org) under the repository ID: FR-FCM-Z3WS. The sequencing data generated in this study have been deposited in the under the associated BioProject, SRA, and BioSample accession numbers are at: https://www.ncbi.nlm.nih.gov/bioproject/PRJNA715957.

Field-specific reporting

Please select the one below that is the best fit for your research. If you are not sure, read the appropriate sections before making your selection.

☐ Life sciences ☐ Behavioural & social sciences ☒ Ecological, evolutionary & environmental sciences

For a reference copy of the document with all sections, see nature.com/documents/nr-reporting-summary-flat.pdf

Ecological, evolutionary & environmental sciences study design

All studies must disclose on these points even when the disclosure is negative.

Study description	The diversity and biogeography of the obligate bacterial predators Bdellovibrio and like organisms in wastewater treatment plants were revealed. Their interactions with prey was identified, first using statistical analyses and confirmed by direct analyses of interacting predators and prey, in separated, sympatric microhabitats (flocs and liquor). Samples were retrieved at 18 time points in at three sites at the same time, in triplicates, and analysis in hierarchical/nested fashions.
Research sample	The research samples were separated in sub-samples of floc and liquor, each in three replicates for each time point and site. Original samples were collected as one liter samples retrieved from activated sludge basins. They were transferred on ice to the lab within hours, and further subjected to chemical analyses and processing. The liquid (bulk water, supernatant) fraction of the activated sludge was separated from its floc fraction by sedimentation on ice for one hour.
Sampling strategy	Each sample consisted of one liter retrieved from the activated sludge basin in each of the plants, at each time point, in triplicates, yielding triplicated samples for each liquor and flocs, at each site and time point. Sample size and time points calculation was performed to enable triplicated sampling and the large number different analyses. This sampling size is sufficient as it enables to statistically discern between origins of the sample while enabling to perform the time series.
Data collection	Data from the samples was obtained by sequencing, qPCR, FACS and chemical analysis. YC and ZP performed the bulk of the sequencing and qPCR analysis, AAR most of the chemical analyses; FS, TH and SM performed the FACS along with YC.
Timing and spatial scale	Sampling was from March 2013 to February 2014, every months except in August and February, one weekly. Data were obtained from all time points for the whole period.
Data exclusions	Samples from March could not be obtained on time from the El-Bireh plant and there were not included.
Reproducibility	Reproducibility of the experiments based on sampling were not possible besides noting the significance of the various statistical analyses performed. In the case of flow cytometry for prey and non-prey attachment, the experiments were performed twice. The testing of prey in dual predator-prey cultures, was performed twice, including three replicates per treatment. The results between experiments were reproducible.
Randomization	Randomization was not relevant to the study as it was hierachical/nested in essence.
Blinding	Blinding was not necessary as no subjective, operator-dependent analysis was performed.

Did the study involve field work? ☒ Yes ☐ No

Field work, collection and transport

Field conditions	The environmental conditions at each time point were recording in detail and are presented in the manuscript, and included: Temperature (bold) (oC); Salinity (ppm); conductivity (µS.m-1); Total suspended solids (TSS) (mg.l-1) Volatile suspended solids (VSS) (mg*l-1); Biological oxygen demand (BOD, (mg.l-1); Chemical oxygen demand (COD, (mg.l-1); Nitrate (NO2,ppm); Nitrate (NO3,ppm); Phosphorus (PO4,ppm); Total Kjehldahl nitrogen (TKN, mg.l-1); Total organic carbon (TOC, mg.l-1); Total dissolved solids (TDS, mg.l-1).
Location	Locations are industrial sites, their names and locations are provided in the manuscript. Therefore, latitude and longitude do not add precision.
Access & import/export	No licenses were required.
Disturbance	No disturbance was inflicted upon the studied sites.

Reporting for specific materials, systems and methods

We require information from authors about some types of materials, experimental systems and methods used in many studies. Here, indicate whether each material, system or method listed is relevant to your study. If you are not sure if a list item applies to your research, read the appropriate section before selecting a response.

Materials & experimental systems

n/a	Involved in the study
☒	☐ Antibodies
☒	☐ Eukaryotic cell lines
☒	☐ Palaeontology and archaeology
☒	☐ Animals and other organisms
☒	☐ Human research participants
☒	☐ Clinical data
☒	☐ Dual use research of concern

Methods

n/a	Involved in the study
☒	☐ ChIP-seq
☐	☒ Flow cytometry
☒	☐ MRI-based neuroimaging

Flow Cytometry

Plots

Confirm that:

☒ The axis labels state the marker and fluorochrome used (e.g. CD4-FITC).

☒ The axis scales are clearly visible. Include numbers along axes only for bottom left plot of group (a 'group' is an analysis of identical markers).

☒ All plots are contour plots with outliers or pseudocolor plots.

☒ A numerical value for number of cells or percentage (with statistics) is provided.

Methodology

Sample preparation	cell fixation (FACS, flow cytometry)
Instrument	BD Influx v7 Sorter USB
Software	FlowJo 10.0.8.r1 (FlowJo, Tree Star / BD); R package flowCybar; R package flowCHIC
Cell population abundance	150,000 cells per gate (P1 and P14) sorted at a sort rate of about 80 cells.sec-1 or 500,000 cells (P16) sorted at a sort rate of about 1,800 cells.sec
Gating strategy	Gates were set for apparent cell clusters. Preliminary experiments were performed by spiking target populations to the environmental samples, providing limits of detection both quantitative (concentration) and qualitative (Fluorescence/FSC detection)

☒ Tick this box to confirm that a figure exemplifying the gating strategy is provided in the Supplementary Information.
